# Do New Vessel Sealing Devices and Harmonic Ace Increase Ureteric Injury in Total Laparoscopic Hysterectomy?

**DOI:** 10.4103/0974-1216.71613

**Published:** 2009

**Authors:** Prakash Trivedi, Sylvia D’Costa, Preeti Shirkande, Meenu Wahi, Shilpi Kumar

**Affiliations:** Professor and Head of Obstetrics and Gynecology Department, Rajawadi Hospital and D Y Patil Medical College, Mumbai, India; 1 Consultant Gynecologist, NILES and Aakar IVF Centre and Holy Family Hospital, Mumbai, India; 2 Clinical Assistant, NILES and Aakar IVF Centre, Mumbai, India; 3 Ex-clinical Assistant, NILES and Aakar IVF Centre, Mumbai, India; 4 ICOG FOGSI Certificate Trainee, NILES and Aakar IVF Centre, Mumbai, India

**Keywords:** Total laparoscopic hysterectomy, ureteric injury, vessel sealing device, harmonic ace

## Abstract

**Objectives:**

To compare the risk of ureteric injury in total laparoscopic hysterectomy (TLH) using new vessel sealing devices (VSDs) and harmonic scalpel with simple scissors, bipolar and suturing. This was an evaluation of 1209 cases, carried out from May 1999 to April 2010.

**Design and Setting:**

A retrospective comparative study was carried out at a tertiary gynecological endoscopic unit.

**Materials and Methods:**

Out of 1209 patients, who had hysterectomies for various indications, TLH was done in 892 patients, 273 had vaginal hysterectomy and 44 had abdominal hysterectomy. We evaluated the incidence of ureteric injury in these cases.

**Results:**

There was no mortality. In the group of vaginal and abdominal hysterectomy, there were no ureteric injuries. In the TLH group, we had 390 cases with simple scissors, bipolar and suturing with no ureteric injury. In 502 cases, new VSDs, e.g., plasma kinetic gyrus, Martin Maxim with Robi grasper, with or without harmonic 5 mm scalpel/ace were used. There were five ureteric injuries, all on the right side (one double ureter): first case was with Martin Maxim and Robi grasper, two with plasma kinetic gyrus 10 mm trissector, one with harmonic scalpel and the last one with scissors. We evaluated the reasons for such ureteric injuries, with experienced laparoscopic surgeons and the best possible set up. There were seven conversions to open surgery out of 892 cases of TLH, more due to poor case selection.

## INTRODUCTION

Any laparoscopic surgeon is concerned about ureteric injury and its profound morbidity, if undetected. The general incidence of ureteric injury, irrespective of the pathology, in abdominal hysterectomy is 1.3–2.2%, in vaginal hysterectomy is 0.03%, and in conventional laparoscopic hysterectomy, it is 1.37%.[1–3]

The close anatomic relationship of lower urinary tract predisposes the distal ureter to risk for injury during pelvic surgery. Intraoperative repair of ureteral injuries is successful in >90% of patients, but if missed, late repair attempt gives poor results.[4] Prophylactic ureteric catheterization has been advocated by some in difficult cases,[5] though it has not been shown to reduce the risk of ureteric damage in routine cases.[6]

Commonly, the ureter is injured in the lower third; two-thirds of ureteric injuries are not recognized intraoperatively. If detected, it can be repaired and renal function is not compromised.[7]

This may change with regular use of vessel sealing devices (VSDs) and harmonic scalpel by an expert or amateur. These new devices may promise less blood loss, less time with improved efficiency, but also have distressing complications. The VSD is fast with a less failure rate for vessel seal. VSD acts by low constant voltage, pulsed high current, and impedance feedback. With high coaptive pressure, 100% hydrogen cross links are first ruptured and good vascular seal is formed.

In harmonic ace, high-frequency vibrations cause a low temperature protein denaturation leading to vessel occlusion by coagulum and cavitational fragmentation.

Maximum temperature for the 2 mm from device varies from 49.9 to 64.5°C.

## MATERIALS AND METHODS

This study was carried out from May 1999 to April 2010 at the National Institute with Laser and Endoscopic Surgery, Mumbai, with all advanced equipments and advanced anesthesia machine with low gas flow, spirometer with multiparameter monitoring - NIBP (Non Invasive Blood Pressure), SPO2 (Saturation Pressure of Oxygen) ETCO2(End Tidal Carbon Di-oxide) ECG (Electro CardioGram) and temperature. The center also has two good bipolars along with VSDs – plasma kinetic gyrus, Martin Maxim and harmonic ace, and five morcellators, three chip cameras, etc.

A total of 1209 patients of hysterectomy were evaluated to actually document the supremacy, if any, of VSDs and harmonic scalpel over regular scissors and bipolar dissection with endosuturing, as needed. In the vaginal or abdominal hysterectomy group, there were no cases of major ureteric injuries. A perfect method for a total laparoscopic hysterectomy (TLH) is an unanswerable debate; innumerable methods have been designed to make it safe and fast, which may add to the cost and complications.

Principles of laparoscopic surgery, anesthesia and position of the patient did not change in both the techniques.

We had patients from 33 to 72 years of age. Hysterectomy was performed for pathologies like fibroids, endometriosis, adenomyosis, ovarian tumor, endometrial carcinoma, etc.

A total of 502 patients had TLH with 10–28 weeks size uterus. Interestingly, there were 39% cases with 1, 2 or 3 previous caesarean sections and 27% cases with uteri more than 18 weeks size. Amongst the new energy sources, plasma kinetic gyrus, Martin Maxim, harmonic scalpel, etc., were used. In 390 patients, TLH was done with simple good bipolar, scissor dissection and endosuturing.

All the patients after necessary preoperative investigations were given peglac on the day prior for bowel preparation.

### Operative techniques

#### Laparoscopic hysterectomy with scissors, bipolar and suturing

Under general anesthesia, patient was placed in the modified lithotomy position. The ports were injected with 0.5% sensorcaine to reduce postoperative pain. Umbilical port was used for the optics (10 mm) and three 5-mm flower valve trocars were inserted at the right Mcburney’s point, at the same point on the left side and the third port was in line with the umbilicus on the left side. This port placement gives ipsilateral control both to surgeon and assistant.

The standard steps for TLH are to release the supports to uterus on both sides, achieve hemostasis with bipolar set at 30–35 W and cut with scissors. Same principle is used if the size of the uterus is big. A simple uterine manipulator of thick hulka’s type and 5 mm myoma screw were used from one of the ports to give direction. The bladder peritoneum was dissected by scissors/monopolar spatula, if needed, and pushed down, small bleeders were taken care of with bipolar, and posterior peritoneum was dissected to see the uterine vessels very well skeletonized. Polyglactin no. 1 was used to suture them on both sides by ipsilateral port suturing. Uterine vessels were separated, at least 1 cm from the origin, to avoid avulsion of the vessels, on delivery of the uterus.

A vaginal tube like McCartney’s designed by the author was used to make a circumferential incision on the vagina. The uterus with pathology was removed vaginally, but more than 14 weeks size was morcellated to avoid vaginal lacerations.

#### Total laparoscopic hysterectomy with vessel sealing device and harmonic scalpel

Anesthesia, position, and port placement were similar, except in the case of bigger uterus and previous vertical scars of CS/pelvic surgery. The primary 10 mm port was 4–5 cm supra umbilical, after inserting a nasogastric tube. The second and the third ports were similar except that they might have been higher. The right-sided port was a lower port in line with the port of the opposite side if 5 mm VSD was used. If 10 mm gyrus VSD was used, the right port was of 10 mm in line with umbilical port.

A 30° 10 mm telescope was kept in the center when dissection was done in the right side and in the right 10 mm port when dissection was taking place on the left side. Quite frequently, we used 5 mm gyrus golden tip coagulator-sealer and cutter in the left port with the harmonic scalpel also on the same side. The myoma screw was put on the opposite side. After the infundibulo-pelvic ligament or cornu, on reaching uterine vessels, the bladder peritoneum was dissected by harmonic scalpel or plasma kinetic golden tip curved coagulator and cutter. Very often, a 10 mm instrument with a small gauze from one of the 10 mm ports was used to push the bladder like in open surgery, with the cut peritoneum held by an atraumatic instrument, making the fibers prominent, and the vaginal tube added the base on which the bladder was pushed down, laterally exposing uterine vessels and safeguarding bladder and ureter. The uterine vessels on both sides were taken care of by the gyrus VSD at two to three points and then cut. After this step, harmonic ace worked best to separate uterine vessels from the uterus for at least 1 cm. We usually cut the uterosacral ligament with harmonic scalpel even before uterine vessels are sealed. This helps the circumferential colpotomy on the silicon tube introduced vaginally. One blade of the harmonic ace was used for a circumferential vaginal incision with a single tooth grasper at the left upper port to guide. Uteri, upto 12–14 weeks size, were delivered vaginally and >14 weeks were morcellated by the Rotocut/Sawahle or power morcellator. The sutures were now used to close the vagina efficiently. After a thorough wash with diluted betadine solution, areas of bleeding were coagulated. In a few cases, a 16 no. Ryle’s tube was kept as a drain which allowed exudation and avoided collection in the pelvis.

The VSD is supposed to make hysterectomy bloodless, painless with good sealing effect and the harmonic scalpel is an excellent dissector. Unfortunately, these increase the cost but it can be avoided by using any suture material for reducing pain and blood loss.

The biggest drawback is the cost of the brain unit. Further, no matter whichever company says that our machine produces less smoke and less lateral spread of heat, these are only marketing techniques. The gyrus has a great advantage that by attaching just the morcellator handpiece to the same brain unit, bipolar morcellation can be done. This way, the cost of a morcellator brain unit can be saved.

The lateral heat spread is present but cannot be scientifically prevented and can lead to ureteric or other injury.

There were five ureteric injuries, all on right side, first case with Martin Maxim and Robi grasper, two with plasma kinetic gyrus 10 mm trissector with one double ureter on the right side, one with harmonic scalpel and the last one with scissors. All the patients were of multiple fibroids with 16–26 weeks size uteri, two had bilateral salpingooopherectomy, and two had previous cesarean sections.

## DISCUSSION

There is no controversy that vaginal hysterectomy is safe, fast and minimally invasive. Abdominal hysterectomy is decreasing, except being used for malignancy and very large uterus. Laparoscopic hysterectomy did start with controversy but has been one of the popular consumer-driven methods of choice.

The equipments for performing TLH changed from 1999 May to April 2010. We have done 390 TLH with simple dissection, good bipolar and suturing. In the last few years, we have done 502 TLH with new VSDs as energy source and harmonic ace as a tissue dissector.

Interesting outcome raises a question “Are these new VSDs and harmonic scalpel cost effective and safe?”

The following observations were made.

1. The cost of a new VSDs and harmonic scalpel brain unit is around Rs. 10–11 lakhs each and the hand instrument, usually disposable, costs between Rs. 16,000/- and Rs. 40,000/- and can be used in three to five cases maximum, though as per company norms it is to be thrown out after one surgery.
2. The new VSD handpiece, either 10 or 5 mm, had a unique advantage of sealing the structures held at the broad tip to achieve hemostasis and has a small switch to cut the tissues with white coagulation and no charring. Thus, we could reach from the infundibulo-pelvic ligament or cornu to uterine vessels, pushing the bladder peritoneum down and almost reaching the vagina for colpotomy fast. Use of only two to three instruments saves time, reduces bleeding and needs no sutures, till reaching closure of the vagina. The harmonic ace is an excellent tissue dissector to cut tissues, ligament, and peritoneum and also helps to push it down for safety of the bladder due to cavitational effect. Especially, in cases of previous two to three cesarean sections, abdominal surgery, large uterus, this combination works very well.
3. The simple TLH with scissors, bipolar and suturing needed multiple changes in instruments, required little extra time, and definitely needed sutures and skills.
4. Our data of 390 simple TLH and 502 TLH with VSD with or without harmonic scalpel showed the following.
  - Time taken by simple TLH was more especially in difficult cases and cases with large uteri, and the ones with previous cesarean section. The blood loss was marginally different. The cost involved in the surgery was much less. There were a few complications like bladder injury which happened in three cases and were sutured laparoscopically. Bleeding was more from the right uterine vessel and below, occasionally necessitating blood transfusion. Conversion to abdominal hysterectomy was rare (1%).
  - In TLH with VSD and harmonic scalpel, the procedure looked simple and faster. We were more comfortable with two 5 mm ports on the left side and two 10 mm ports one in the center for optic and other on the right side in same line laterally. Only a few instruments were used – gyrus 10 mm VSD with trissecter cutting, harmonic ace, a simple fenestrated atraumatic grasper and a 5-mm or 10 mm myoma screw for uterine manipulation.

Among the 502 cases of TLH with VSD and harmonic scalpel, we had five ureteric injuries, for the first time in the last 25 years of practice. Each case was with a different VSD and harmonic scalpel and the last one had a simple scissors dissection. Bladder injury was only in one case. Bleeding in two patients necessitated blood transfusion.

**Figure 1:**
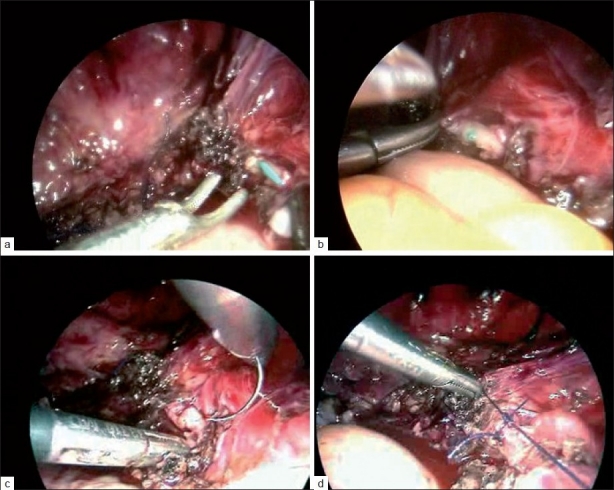
(a) Cystoscopic right ureter, (b) two ends of ureter brought closer over stent, (c) laparoscopic uretero-ureteric stenting, (d) uretero-ureteral anastomosis

On detailed analysis, we found that the last 502 cases had much larger uterus >15 weeks size, till 28 weeks, and had previous cesarean sections which itself could be contributory to injuries. The second reason was that due to the ease and comfort with which we could use the VSD and harmonic ace, we tend to overuse them, occasionally close to vital structure. The injury with scissors was seen and intraoperative D-J stenting and laparoscopic uretero-ureteral anastomosis were performed [Figure 1a–d]. The stent was removed later and the patient was fine.

**Figure 2:**
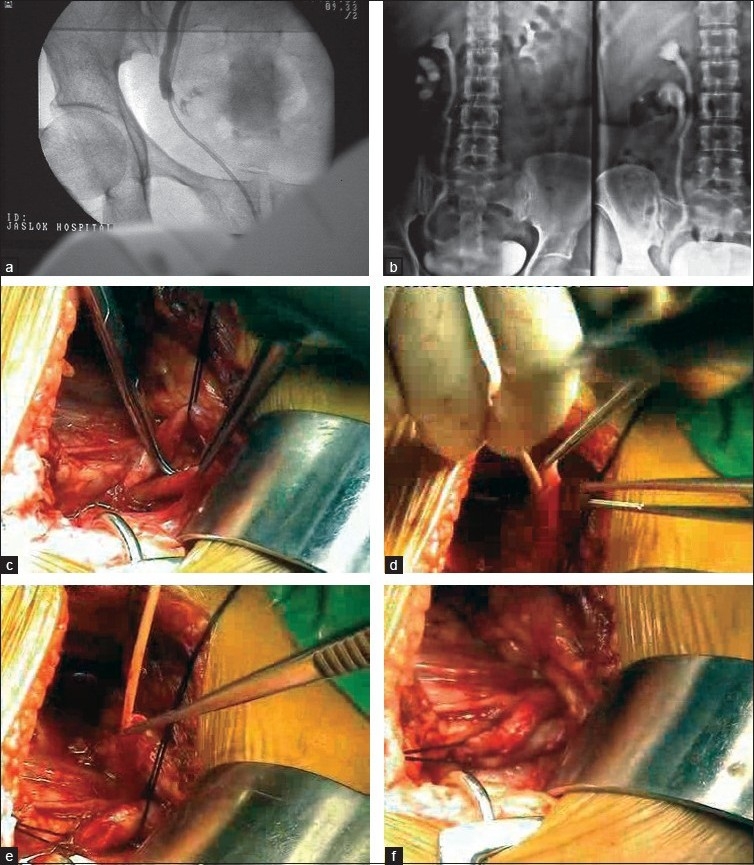
(a) Retrograde pyelography, (b) IVP showing double ureter on the right side, (c) right double ureter, (d) injured ureter stented, (e) injured ureter brought closer to the other ureter, (f) uretero-ureteral anastomosis completed

**Figure 3:**
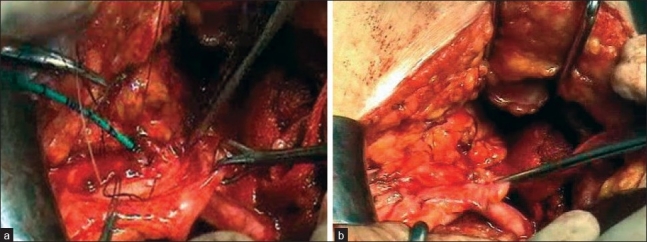
(a) After making fish mouth neocystostomy and (b) ureterovesical anastomosis were done

Of the two patients needing open surgery, one had a right-sided double ureter with the upper moiety draining low in the bladder next to vagina (injured) and the lower moiety draining up [Figure 2a–c]. The uretero-ureteral anastomosis, i.e., one ureter draining in the other was done [Figure 2d–f]. The patient fared well. In the remaining three cases requiring open surgery, the lower end of the ureter had a fish mouth and neocystostomy and ureterovesical implantation were done [Figure 3a and b].

One patient was lost to follow up. None of the patients with ureteric injury were charged for the second surgery.

On further discussion with the urologist, we were advised not to underestimate the lateral heat spread with any of this modality and make it compulsory to trace the ureters on both sides after a TLH is done with VSD and harmonic scalpel. A drainage tube can give clue of urine leak in the peritoneum.

## CONCLUSION

In this study carried out from May 1999 to April 2010, we evaluated the role of VSDs and harmonic scalpel in TLH (502 cases), and a simple TLH with scissors, bipolar and endosuturing (390 cases), in the incidence of ureteric injury. There were 44 abdominal hysterectomies and 273 vaginal hysterectomies done out of 1209 hysterectomies done in the last 11 years.

The conclusion is that the ureteric injury was not seen in our practice from 1985 till 2005 in any form of hysterectomy. Both vaginal and abdominal hysterectomies were fine. All the 309 cases, who had simple TLH with scissors, bipolar and suturing, did not have any ureteric injury.

From 2005, we had five cases of ureteric injuries, all on the right ureter with one case of right double ureter; intraoperative DJ stent and uretero-ureteral anastomosis were done in one case with the injury by scissors.

The other four ureteric injuries were by different new VSDs and harmonic scalpel, were detected later and treated in time to save the kidney function.

The major contributory factors for ureteric injury were difficult, large uteri, patient with previous cesarean, abdominal surgery and transient overconfidence of the surgeon to the new gadgets without titrating the lateral damage. The VSDs and harmonic scalpel have their own benefits. Any new instrument has a phase of excitement, euphoria, overconfidence, troubles, and then solutions.

Most important aspect of advanced technology is that a good surgeon should identify the complication, counsel the patients and relatives, giving the best possible treatment which makes you stand apart from any other endoscopist. Also, simple skeletonization of uterine vessels and good suturing are still better with any endoscopist.
